# High maternal BMI and low maternal blood BDNF may determine the limit of detection of amniotic fluid BDNF throughout gestation: Analysis of mother-fetus trios and literature review

**DOI:** 10.1371/journal.pone.0265186

**Published:** 2022-03-10

**Authors:** Anne Flöck, Alexandru Odainic, Ramona Dolscheid-Pommerich, Michael Robert Jaskolski, Anna Katharina Diedrich, Marie Therese Puth, Susanne Schmidt, Birgit Stoffel-Wagner, Brigitte Strizek, Ulrich Gembruch, Waltraut Maria Merz

**Affiliations:** 1 Department of Obstetrics and Prenatal Medicine, University Bonn Medical School, Bonn, Germany; 2 Institute of Innate Immunity, University of Bonn, Bonn, Germany; 3 Department of Microbiology and Immunology, The Peter Doherty Institute for Infection & Immunity, University of Melbourne, Victoria, Australia; 4 Institute of Clinical Chemistry and Clinical Pharmacology, University Bonn Medical School, Bonn, Germany; 5 Department of Medical Biometry, Informatics and Epidemiology, University Bonn Medical School, Bonn, Germany; Holbaek Sygehus, DENMARK

## Abstract

**Objective:**

An increasing number of studies show the importance of brain-derived neurotrophic factor (BDNF) acting at the feto-placental interface, however, only a few studies describe BDNF levels in amniotic fluid (AF).

**Methods:**

In this cross-sectional, prospective study, 109 maternal blood-amniotic fluid pairs (including 66 maternal blood-fetal-blood-amniotic fluid trios) were analyzed. BDNF concentrations were measured with a commercially available immunoassay.

**Results:**

In 71 AF from 109 samples, AF-BDNF concentrations were below the lowest limit of Quantitation (LLoQ) of 1.19 pg/ml (group A), leaving 38 samples with measurable BDNF concentrations (group B). Patients in group A showed significantly higher maternal BMI before pregnancy (mean±SD 26.3± 6.7 (kg/m^2^) vs. 23.8 ±4.5 (kg/m^2^) p = 0.04) and lower maternal blood BDNF concentrations than the other group (mean±SD 510.6 ± 554.7 pg/ml vs. mean±SD 910.1± 690.1 pg/ml; p<0.0001). Spearman correlation showed a negative correlation between maternal BMI before pregnancy and maternal BDNF concentrations (r = -0.25, p = 0.01).

**Conclusion:**

Our study is the first to correlate AF-BDNF samples with the corresponding maternal and fetal blood-BDNF samples. The significant negative correlation between maternal BMI before pregnancy and maternal BDNF and AF-BDNF concentrations below the limit of detection has to be evaluated in further studies.

## Introduction

Brain-derived neurotrophic factor (BDNF) is involved in energy metabolism [1,2] and various CNS functions [3,4]. Reduction in brain-derived neurotrophic factor (BDNF) expression in the brain as well as mutations in BDNF gene or its receptor are associated with obesity in human and animal models [5]. An increasing number of studies show the importance of BDNF acting at the feto-placental interface and a key role for BDNF in fetal growth and development is assumed [6–8]. During pregnancy, BDNF promotes implantation and placental development [9]; levels rise with gestational age (GA) [10,11]. Investigations in mice and rats suggest that maternal BDNF reaches the fetal brain through the utero-placental barrier and may contribute to its development [12–14].

Amniotic fluid (AF) is a complex composition of fetal and maternal fluid and cellular components. The fluids are regulated by fetal urine, lung fluid and swallowing, while intramembranous transport allows exchange between AF and fetal blood or the uterine wall [15,16]. Cellular components in AF are heterogenous and still poorly understood [17,18]. Mainly consisting of fetal skin and urothelial cells, maternal neutrophils have also recently been shown to invade the amniotic cavity [19]. BDNF in maternal serum predominantly reflects levels in platelets [20], but the source of AF-BDNF is unknown. In adults, BDNF has been found in urine of healthy individuals, but likewise its source has yet to be identified [21,22].

Reference values in human maternal and cord blood throughout gestation have not yet been established and only a small number of studies to date have analyzed BDNF levels in amniotic fluid (AF) [23–26]. These studies present AF-BDNF concentrations exclusively from amniocenteses (AC) during the late first to the early second trimester and do not provide information about maternal or fetal BDNF levels. The aim of our study was to analyze BDNF concentrations in AF throughout gestation and to correlate maternal and fetal BDNF blood concentrations with AF-BDNF concentrations. To adjust for a potential dilutional effect, the amniotic fluid-BDNF/amniotic fluid-total protein ratio (AF-BDNF/AF-TP) was analyzed.

## Material and methods

### Data collection

This cross-sectional, prospective study was performed between August 2019 and December 2020 in the obstetric unit of a tertiary referral center. Study design and consent forms were approved by the institutional ethics committee (No. 305/11). All women gave their written informed consent. Baseline demographic and clinical data, antenatal history, including pregnancy complications, obstetric, delivery and newborn data, were obtained from the patient charts and the departmental perinatal database.

All patients with singleton or twin pregnancies presenting to the ward for diagnostic amniocentesis, fetal therapy (laser coagulation of fetal anastomoses in twin-to-twin transfusion syndrome (TTTS), fetal tracheal occlusion with balloon catheter (FETO)) or before C/S were included. Exclusion criteria were unwillingness to take part in the study, higher order multiple pregnancies, premature rupture of membranes, blood-stained AF samples, mothers with clinical signs of infection (fever >38°Celsius, leukocyte count > 15 G/l).

Sample size of the study was started with the aim to analyze AF-BDNF concentrations throughout different weeks of gestation in about 250 patients during 2 years. Oriented at the sample size of existing literature on AF-BDNF and because the results after one year revealed a lack of detection of AF-BDNF in the majority of cases, the collection and analysis of AF-BDNF samples was stopped at 121 cases to evaluate possible reasons for this fact.

### Ultrasonographic evaluation

Ultrasonographic and echocardiographic evaluation was performed only by experienced sonographers with high-resolution ultrasound equipment (Philips EpiQ7, Philips Hamburg, Germany; Voluson E10, GE Munich, Germany, Toshiba Aplio 900, Canon Medical Systems, Neuss, Germany) in all cases, with 5–9 and 2–6, 7, and 8 MHz convex transducers, respectively. A detailed assessment of the fetal anatomy and cardiovascular status including echocardiography and Doppler examination was performed in all subjects. Estimated fetal birth weight (EFW) was calculated [27] and fetal growth restriction (FGR) defined as EFW <10th percentile for GA. Sonographic amount of AF was calculated by measuring the deepest vertical pocket (DP) of amniotic fluid. Cases with DP <2cm were classified as oligohydramnios, cases with DP >8cm were classified as polyhydramnios.

### Blood sample analysis

Maternal venous blood samples (6 ml) were taken on admission immediately before fetal therapy, amniocentesis or cesarean section. AF samples (3–15 ml) were taken during diagnostic amniocentesis, fetal therapy (laser coagulation of fetal anastomoses in twin-to-twin transfusion syndrome (TTTS), fetal tracheal occlusion with balloon catheter (FETO)) or before C/S. Blood-stained samples were discarded. Fetal blood samples were taken either as newborn venous blood samples (6 ml) from the placental part of the umbilical cord, immediately after clamping of the cord, and before delivery of the placenta, or during fetal blood sampling (1-3ml) at feticide, after percutaneous puncture of the umbilical vein, before potassium chloride injection.

All samples were transferred to +4°C immediately until centrifugation (4 000 rpm, 10 min.); thereafter, serum was separated and stored at -80°C.

AF-BDNF concentrations were quantified according to the manufacturer’s protocol with the human BDNF Simplex ProcartaPlex Kit (Thermofisher, Germany, catalogue number EPX01A-12116-901, assay detection limit 1.02 pg/ml). Samples were only thawed once. Thawed samples were centrifuged again at 9700 rpm at 4° C for 10 min. Fluorescence intensities for BDNF were analyzed with the FLEXMAP 3D System (Luminex, Netherlands). The mean fluorescence intensities (MFIs) of the standard dilution series were fitted to a five parameter linear weighted curve to determine the BDNF concentration of each serum sample and normalized by the Milliplex Analyte software (v5.1.0.0). Limit of detection was 1.19 pg/ml.

Amniotic fluid total protein (AF-TP) concentrations were analyzed in batches at the institutional laboratory of the University Hospital Bonn. Total protein was determined turbidimetrically (cobas c702, Roche Diagnostics, Mannheim, Germany). To adjust for a potential dilutional effect, the AF-BDNF/AF-TP ratio was analyzed in samples with measurable BDNF.

### Statistical analysis

For data analysis, the statistical software package R Core Team (2020; version 4.0.2, R: A language and environment for statistical computing. R Foundation for Statistical Computing, Vienna, Austria. URL https://www.R-project.org/) was used. Between group comparisons of approximately normally distributed variables were performed by analysis of variance or t-tests for independent samples, otherwise Kruskall-Wallis or Mann-Whitney-U test was used. p<0.05 was considered statistically significant.

## Results

### Baseline characteristics

In total, 121 amniotic fluid samples (n = 121) and their corresponding maternal blood samples were collected. Of these, 12 fetal blood-amniotic fluid pairs were excluded because of missing maternal blood samples, leaving 109 maternal blood-AF pairs (including 66 maternal blood-fetal-blood-AF trios) for analysis. These 109 pairs constitute the study group. Fifty-seven fetal blood samples were taken during C/S, and nine during fetocide. In 71 from 109 samples (group A), AF-BDNF concentrations were below the LLoQ of 1.19 pg/ml. Only in 38 patients (group B) AF-BDNF concentrations were above the limit of detection.

Demographic, obstetric, and sample processing data are listed in “**Table 1”**.

**Table 1:** Maternal, fetal and sample processing data of group A (AF-BDNF not detectable) and group B (AF-BDNF detectable), (n = 109).

	Group A n = 71	Group B n = 38	p value
**Maternal data:**			
**Age** (years) mean±SD	33.5±5.2	32.8±5.4	0.504
**BMI before pregnancy** (kg/m^2^) mean±SD Missing	26.3±6.7 3	23.8±4.5 1	0.042
**BMI before pregnancy_ groups** (kg/m^2^) <30 30–34.9 >35 Missing	53 (77.9%) 7 (10.3%) 8 (11.8%) 3	34 (91.9%) 1 (2.7%) 2 (5.4%) 1	0.264
**platelet count** (n/μl) mean±SD Missing	221.5±55.8 6	213.4±67.5 4	0.550
**BDNF serum concentration** (pg/ml) mean±SD	510.6±554.7	910.1±690.1	<0.001
**Adverse pregnancy outcomes:**			
**preeclampsia (PE)**	2 (2.8%)	2 (5.3%)	0.610
**gestational diabetes (GDM)**	13 (18.3%)	3 (7.9%)	0.238
**Fetal growth restriction FGR** (fetal birth weight <10. percentile)	8 (11.3%)	7 (18.4%)	0.510
**Fetal data:**			
**BDNF serum concentration** (pg/ml) mean±SD	n = 41 364.3±295.7	n = 25 449.0±309.9	0.200
**Gestational age (GA)** (weeks) mean±SD	32.2±8.5	31.0±8.6	0.739
**Indication for AC** -diagnostic AC -TTTS Laser (recipient) -FETO -fetocide -C/S	10 (14.1%) 12 (16.9%) 2 (2.9%) 5 (7%) 42 (59.2%)	7 (18.4%) 6 (15.8%) 1 (2.6%) 4 (10.5%) 20 (52.6%)	0.949
**singletonstwins** • MCDA • DCDA	51 (71.8%) 20 (28.2%) 13(65%) 7 (25%)	31 (81.6%) 7 (18.4%) 6 (85.7%) 1 (14.2%)	0.373
**ultrasound anomalies** Missing cardiac thoracic cerebral abdominal skeletal renal multiple/syndrome	16 (22.8%) 1 7 (43.8%) 3 (18.8%) 0 1 (6.3%) 0 2 (12.5%) 3 (18.8%)	16 (42.1%) 0 1 (6.3%) 5 (31.3%) 4 (25.0%) 1 (6.3%) 2 (12.5%) 0 3 (18.8%)	0.061
**karyotype** not available available, normal available, abnormal	54 (76.1%) 15 (21.1%) 2 (2.8%)	22 (57.1%) 12 (31.6%) 4 (10.5%)	0.073
**sample processing data:**			
**time between** **sample collection and centrifugation** (hours) mean±SD Missing	39.6±58.7 7	34.8±48.0 11	0.781
**AF-TP concentration** mean±SD	2256.2±1769.0	2445.2±1891.1	0.753
**AF-BDNF concentration** (pg/ml) mean±SD	-	9.5±3.7	-

total protein; BMI, body mass index; c/s, cesarean section; DCDA, dichorionic diamniotic twins; FETO, fetal endoscopic tracheal occlusion; GA, gestational age; MCDA, monochorionic diamniotic twins; SD, standard deviation; TTTS, twin-to-twin-transfusion-syndrome.

More than 95% of patients were of caucasian ethnicity. In group A and in group B, one patient had a history of psychiatric disorder (bipolar disorder and panic disorder, both stable for years without medication). Four patients developed pregnancy-induced hypertension or preeclampsia (n = 2 group A, n = 2 group B) and 16 patients developed gestational diabetes (n = 13 group A, n = 3 group B). Furthermore the rate of FGR (fetal birth weight <10.percentile) was equally distributed (n = 8 in group A, n = 7 in group B). None of the parameters showed significant differences between groups A and B.

In over 80% of cases the pregnancy ended with one living fetus. In 13 cases the fetus died at fetocide, because parents opted for termination of pregnancy (A: n = 5, B n = 8); in 4 cases one fetus died during pregnancy after TTTS laser therapy; (A:n = 3, B: n = 2); in 3 cases the newborn died after birth (A: n = 1 birth at 24 weeks of gestation with intraventricular hemorrhage, B: n = 2 due to lung-hypoplasia because of congenital diaphragmatic hernia).

### Comparison between groups A and B

**Fig 1:**
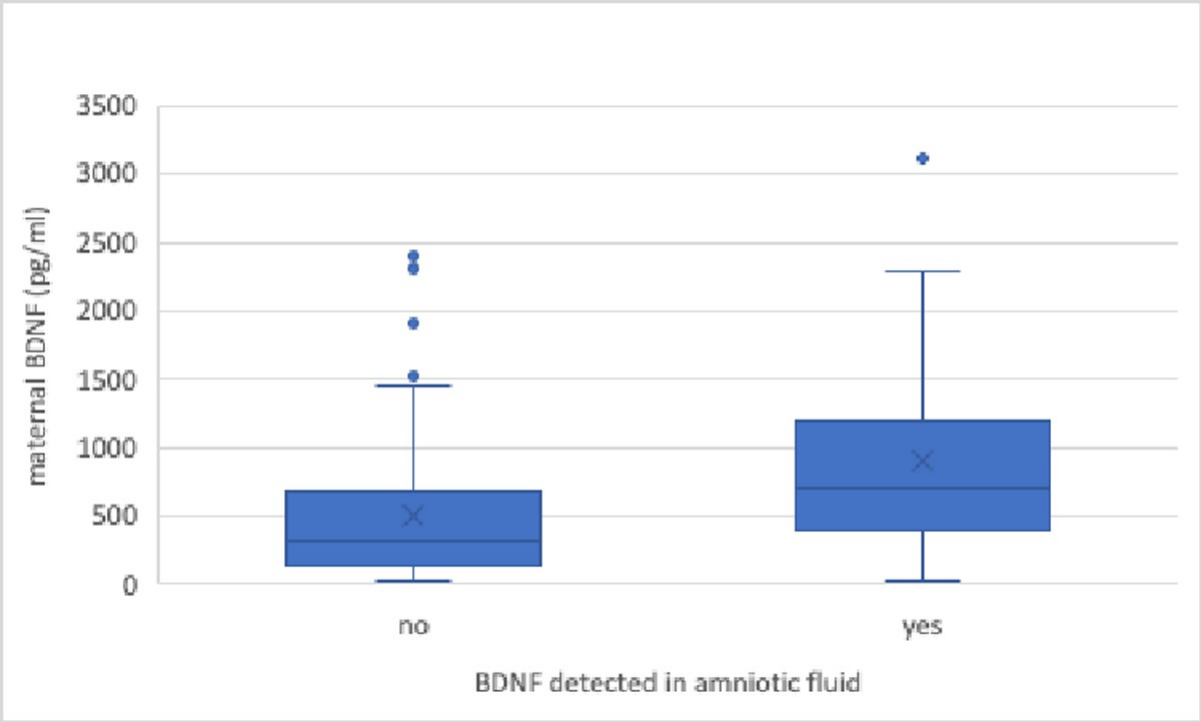
Maternal Blood BDNF concentration (pg/ml) in cases with and without detectable AF- BDNF (group A and B). In specimens of group A, BMI before pregnancy was significantly higher (mean±SD 23.8 ± 4.5 (kg/m^2^) vs. mean±SD 26.3 ± 6.7 (kg/m^2^); p = 0.042), with a significant correlation between maternal BMI before pregnancy and maternal BDNF concentrations (r = -0.25; p = 0.01). “**S3 Fig”**.No significant difference was seen comparing maternal, fetal an AF-BDNF between obese (BMI≥30 kg/m^2^) and non-obese (BMI<30 kg/m^2^) patients (maternal BDNF p = 0.06, fetal BDNF p = 0.08, AF-BDNF p = 0.08). No difference was found between group A and B regarding maternal age, smoking status, maternal platelet count, GA, indication for amniocentesis, abnormal karyotype, fetal anomalies, ultrasound estimation of AF volume, twin rates and chorionicity, AF-TP concentration, and time interval between sample collection and centrifugation **“Table 1”**.

Maternal BDNF blood concentrations were significantly higher in AF specimens of group B (median (IQR) 317.8 (537.1) pg/ml vs. 705.8 (759.2) pg/ml; p<0.001; “**Fig 1**”, with a significant correlation between maternal blood BDNF concentrations and AF-BDNF/AF-TP ratio (r = 0.35, p = 0.0001). “**S1 Fig”.** Additionally, a significant correlation between AF-BDNF/AF-TP ratio and GA was present (r = 0.31, p = 0.023). “**S2 Fig”**.

### Analysis of mother-fetus-AF trios

**Fig 2:**
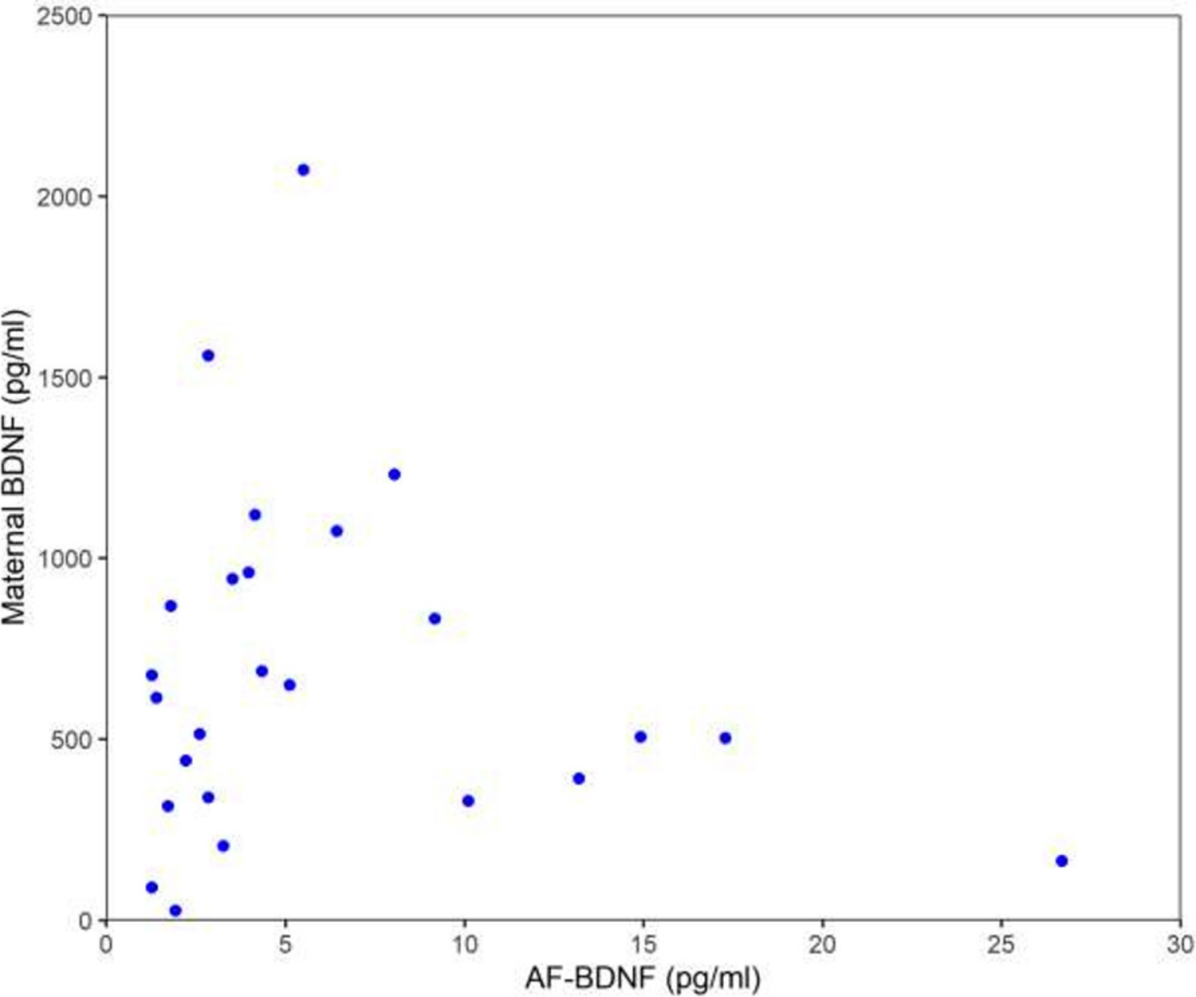
Spearman correlation between maternal blood BDNF and AF-BDNF (pg/ml); r = 0.36, p = 0.003, n = 66; n = 41 samples below the limit of detection (1.19 pg/ml). Maternal and fetal blood BDNF concentrations showed a weak correlation (r = 0.27, p = 0.03). **“S4 Fig”**.

For mother-fetus-AF trios (n = 66), a significant correlation was found between maternal blood BDNF concentrations and AF-BDNF/AF-TP concentrations (r = 0.36, p = 0.003), whereas no correlation was found between fetal blood and AF-BDNF/AF-TP concentrations. **“Fig 2”.** In **“Fig 2”** samples below the limit of detection (1.19 pg/ml) n = 41 were excluded due to better visibility.

## Discussion

### AF-BDNF in literature

Our study is the first to analyze BDNF concentrations in a large cohort of uncultured AF samples at different GA ranging between 15 and 41 weeks, and the first to correlate AF samples with the corresponding maternal and fetal blood samples. Studies on BDNF concentrations in AF are limited to data from AC during a narrow time interval–i.e., late first to early second trimester. Marx et al. [23] were the first to report about 134 AF samples obtained at routine AC at around 17 weeks of pregnancy and were able to detect BDNF in all undiluted samples. In contrast to our data, they found a modest but significant negative correlation between AF- BDNF and GA.

However, data comparison is difficult because information about AF volume is lacking. AF-samples of the studies in **“Table 2”** presented various indications for AC including abnormal pregnancies/fetuses. Those pregnancies are at higher risk to have dysregulations of AF-volume. In our group B, GA was moderately positively correlated with AF-BDNF/AF-TP ratio. In 2010, Cattaneo et al. [24] found no effect of gestational age on BDNF levels in 139 cultured AF samples obtained at routine AC between 15 and 17 weeks of gestation in healthy mothers and fetuses with normal karyotype. Furthermore, the authors were able to show that samples containing the BDNF Val66Met polymorphism had significantly lower BDNF levels in AF. Due to our study protocol, we could not confirm these findings or prove whether the BDNF Val66Met polymorphism or interference/formation of heterodimers with other neutrophins is the reason for the lack of BDNF detection in the majority of our samples. Additionally, we used uncultured AF samples. In 2018, another two studies [25,26] described BDNF concentrations in AF samples obtained at 14–22 weeks of gestation and correlated these with fetal or maternal clinical variables after birth. Antonakopoulos et al. [25] described a need for 20-fold dilution prior to the assay as BDNF levels in AF were thousandfold higher than described in our and other studies **“Table 2”.**

**Table 2:** Literature on BDNF in human amniotic fluid.

Name/year	Sample dilution	Assay	Limit of detection	n/GA	mat. weight/BMI	Indication for AC	Abolute levels	results
**Marx 1999 [23]**	undiluted	Promega Inc. (Germany)	7.8 pg/ml	n = 102 healthy Mean GA 17.6±2.2 weeks N = 13 fetal abnormalities ->N = 6 CNS abnormalitiy ->N = 5 intrauterine infection ->n = 2 chromosomal abnormality	no information	n = 77 Age; n = 17 history of birth defects; n = 10 ultrasound abnormality; n = 48 abnormal serum screening: n = 8 others	not mentioned	-BDNF decreased with GA (r = -0.21) - lower levels in patients with maternal infection during pregnancy -no association with age/ethnicity -recovery studies indicate that actual concentrations in human AF are higher than those measured by ELISA
**Cattaneo 2010 [24]**	not mentioned	R&D Systems (USA)	20 pg/ml	N = 139 15–17 weeks’ AC Mean GA16.1±1.9 weeks	no information	maternal age Infections/ abnormal karyotype excluded	Met carriers 10.02±9.62 pg/ml Non carrriers 15.88±12.71 pg/ml	-lower BDNF in Met allele carriers -no effect of GA, age, fetal sex
**Antonakopoulos 2018 [25]**	at least 20-fold	R&D Systems (USA)	0.372–1.35 pg/ml	N = 80 31 SGA 31 AGA 18 LGA 15–22 weeks’ AC	no difference in maternal weight	Various indications, details not presented	SGA:36300±9000 pg/ml AGA:32700±11200 pg/ml LGA:35700± 5700 pg/ml	-SGA and LGA fetuses with higher BDNF compared to AGA fetuses -correlation between growth restriction and levels
**Deuschle 2018 [26]**	1:2	Promega Inc. (Germany)	0.7pg/ml	N = 79 Singleton, healthy, AGA Mean GA at AC 15.9±0.9 (14.3–18.9 w) Maternal conditions: n = 19 goiter n = 4 hypertension n = 1 congenital Heart disease n = 1 coagulation disorder	no information	n = 61 age-related risk n = 5 abnormal First-trimester screening n = 3 abnormal first-trimester ultrasound	165.1±109.1 (25.8–598.4) pg/ml	-no difference between 2 different timepoints of transport at room temperature to lab (19-23h vs. 30-75min) -CTQ correlated -no correlation with psychiatric disorders/perceived stress, AC-induced stress, anxiety -positive correlation with ELS

AC, amniocentesis; AGA, appropriate for gestational age; BDNF, brain derived neurotrophic factor; CTQ, Childhood Trauma Questionnaire; CNS, central nervous system; ELS, early life stress; GA, gestational age; LGA, large for gestational age; SGA, small for gestational age.

In our opinion, interpretation and comparison of the results of the existing studies is not possible due to insufficient knowledge about the exact origin of BDNF in urine of adult individuals and amniotic fluid. Absent reference values according to GA, different ELISA assays used (no standardized calibrations for assays are available) and heterogeneity of patients and sample preparation are other factors which hinder establishing of hypotheses. An additional complexity arises from the dynamic changes of AF composition and volume during pregnancy and in certain pregnancy complications, such as oligo- or polyhydramnios. The other studies mentioned above failed to take that into account. To adjust for a potential dilutional effect and to make studies comparable in future, we think the AF-BDNF/AF-TP ratio should be provided and further studies are needed to prove a possible association between maternal obesity and AF-BDNF levels, before AF-BDNF could be seen as a possible biomarker in clinical practice. To adjust for a potential dilutional effect, the AF-BDNF/AF-TP ratio should be provided.

### Possible origins of AF-BDNF synthesis

Amniotic fluid (AF) is a complex composition of fetal and maternal fluid and cellular components. Cellular components in AF are heterogenous and still poorly understood [17,18]. Mainly consisting of fetal skin and urothelial cells, maternal neutrophils have been shown to invade the amniotic cavity [19]. BDNF in maternal serum predominantly reflects levels in platelets [20], but the source of AF-BDNF is unknown. In healthy adults, BDNF is found in urine at levels around 110 pg/ml without any circadian variation or difference between genders [22], but its source is also not known. In our study, mean AF-BDNF concentrations (group B) were 3.3 ± 11.2 pg/ml. Interestingly, in rats, BDNF mRNA is upregulated postnatally in the urinary bladder from day 5 to 15 and decreases afterwards to almost undetectable levels [21,28]. However, it is unclear whether these facts are applicable to humans.

Our study showed a positive correlation between maternal blood BDNF concentrations and AF-BDNF concentrations, while there was no correlation with fetal blood BDNF values, indicating a maternal or placental rather than a fetal source of AF- BDNF.

### Correlation between AF-BDNF and maternal or fetal blood

To our knowledge, this is the first study to correlate AF samples with the corresponding maternal and fetal blood samples in 109 maternal blood-AF pairs including 66 maternal blood-fetal-blood-AF trios. BDNF in maternal serum predominantly reflects levels in platelets [20], but the source of fetal BDNF has to be elucidated. BDNF is expressed in human fetal ovary during all stages of ovarian development at weeks 9–20 of gestation [29] and several studies reported on levels in cord blood [30]. We found only five studies reporting on maternal and cord blood together [10,11,31–33] and all of them found higher BDNF concentrations in maternal compared to cord blood and only a weak positive correlation between maternal and fetal blood [10,30]. Furthermore, Dingsdale et al. [30] have shown in a mouse model, expressing BDNF by megakaryocytes, that maternal BDNF does not cross the placenta. Chow et al. [34] described estradiol, multiple microRNAs and post-translational processing of precursor proteins and intracellular shuttling as regulators of BDNF expression in different maternal and fetal tissues. Whether these factors influence the expression of cord or AF-BDNF and how they affect fetal development physiology has to be elucidated.

In summary, the weak correlation between maternal and fetal blood BDNF values and the significant correlation between maternal blood BDNF and AF-BDNF/AF-TP concentrations suggest a maternal or placental source of AF-BDNF and confirm the hypothesis that cord blood levels reflect mainly a fetal synthesis of BDNF.

### Correlation between AF-BDNF and pre-pregnancy BMI

Reduction in BDNF expression in the brain as well as mutations in BDNF gene or its receptor are associated with obesity in human and animal models [5]. Prince et al. [35] suggested that maternal obesity adversely influences BDNF binding and induction of TRKB phosphorylation in placentas from female fetuses. However, the association between circulating levels of BDNF and obesity remains undefined and is based on the assumption that circulating BDNF mirrors concentrations in the brain [5]. We were able to show a significant negative correlation between maternal BMI before pregnancy and maternal BDNF. We also showed higher mean maternal BMI and lower mean maternal blood-BDNF in cases without detection of AF-BDNF. However no significant difference was seen comparing maternal, fetal an AF-BDNF between obese and non-obese patients. A fact that could be attributed to the problem that maternal BMI alone does inevitably not always reflect obesity and the number of severe obese during pregnancy and sample sizes especially of obese patients in group B were small. Interestingly in the group of severe obese patients (BMI >35) we had n = 8 in group A including N = 4 with BMI >40 and only (n = 2) in group B with BMI between 35–40.

Regarding the studies about amniotic fluid and BDNF, only Antonakopoulos et al. [25] provided information about maternal weight. They did not find differences between the groups. In contrast to our results, the meta-analysis of Sandrini et al. [5] found no association between obesity and circulating BDNF concentrations in plasma and serum in non-pregnant patients in ten included studies.

### Hypotheses for the lack of detection for AF-BDNF

BDNF concentrations in plasma are much lower than in serum, while serum BDNF is more stable, its amount is modified by temperature and time of clotting [36] Plasma BDNF is influenced by several clinical factors, such as, inter alia, body weight [37] and circadian rhythm [35] and is sensitive to preparation procedure [38–40]. We can only speculate that AF-BDNF acts like human plasma BDNF and we did not find differences between group A and group B apart from maternal pre-pregnancy BMI. Due to our study protocol, we were not able to elucidate whether the BDNF Val66Met polymorphism or interference/formation of heterodimers with other neutrophins are the reason for the lack of BDNF detection in the majority of our samples. Also, we used uncultured AF samples. Despite well-established use of human amniotic fluid cells (AFCs) in routine prenatal diagnostics, little is known about their origin and properties. Prusa et al. [18] described that fresh untreated and uncultivated human AF samples did not contain cells with neuronal-like morphologic conditions that express markers for neuronal stem and progenitor cells, like BDNF. Only cultivation in standard medium resulted in up-regulation of neuronal markers in human AF samples and Western blot analyses proved BDNF protein expression. In 2017, Gomez-Lopez et al. [41] were able to show by DNA fingerprinting of single AC samples that amniotic fluid neutrophils can be a mixture of fetal and maternal origin. Neutrophils, are a source of BDNF [42] and they increase during late gestation. The decidua in pregnancy is a highly complex tissue containing unique, highly specialized leukocyte subpopulations for each stage of GA with potential to secrete a variety of cytokines [43]. Maternal neutrophils therefore may invade the amniotic cavity and become a source of cytokines.

The following hypotheses can be reached from the BDNF concentrations below the limit of detection in our cohort: a) In our study, uncultivated human AF samples were used. The lack of BDNF detection may be due to inactivated state of AF cells. b) Especially in cases with low maternal blood BDNF concentrations, AF-BDNF is probably reflecting a lower proportion of activated maternal neutrophils in the amniotic cavity, rather than of fetal origin of neutrophils. c) The moderate correlation of AF-BDNF/AF-TP ratio with gestational age may reflect the increase of maternal neutrophils into the maternal-fetal interface during late gestation and could be the main source of AF BDNF.

## Limitations

We are presenting a heterogenous group of patients and fetuses in a cross-sectional study design. Because indication for amniocentesis and fetal blood sampling during pregnancy is limited to fetal diseases due to the risks posed by the intervention and since there has been a decline of diagnostic amniocenteses in recent years, we think it is feasible to include these samples into our analysis. We incorporated a majority of healthy mothers and fetuses and assume our findings to be valid. The lack of standardization of BDNF assays is a general limitation which hampers comparisons of studies. Furthermore the small sample size of certain subgroups may be an explanation, why differences in distribution did not reach statistical significance.

## Conclusion

Our study is the first to correlate BDNF in AF samples with the corresponding maternal and fetal blood samples in a large cohort of uncultured AF samples at different gestational ages. The positive correlation between maternal blood BDNF concentrations and AF-BDNF concentrations, and the lack of correlation between maternal and fetal blood BDNF values indicate a maternal or placental source of AF-BDNF. Besides that, we were able to show a significant negative correlation between maternal BMI before pregnancy and maternal BDNF, and a lack of detection of AF-BDNF in cases with higher maternal BMI and low maternal blood-BDNF that has to be evaluated in further studies.

## Supporting information

S1 Dataset Dataset on AF-BDNF. (SAV)

S1 Fig Correlation between maternal BDNF and AF-BDNF/AF-TP ratio (r = 0.35, p = 0.0001). (TIFF)

S2 Fig Correlation between AF-BDNF/AF-TP ratio and gestational age (r = 0.31, p = 0.023). (TIFF)

S3 Fig Correlation between maternal BMI before pregnancy and maternal BDNF (r = -0.25, p = 0.01). (TIFF)

S4 Fig Correlation between maternal BDNF and fetal BDNF (r = 0.27, p = 0.003). (TIFF)

## Abbreviations

AF: amniotic fluid
AF-BDNF/AF-TP ratio: amniotic fluid-BDNF/amniotic fluid-total protein ratio
BBB: blood-brain barrier
BDNF: brain-derived neurotrophic factor
C/S: cesarean section
DCDA: dichorionic diamniotic twins
DP: deepest vertical pocket of amniotic fluid
EFW: estimated fetal birth weight
FGR: fetal growth restriction
GA: gestational age
LLoQ: Lowest Limit of Quantitation
MCDA: monochorionic diamniotic twins
MFI: mean fluorescence intensities
MoD: mode of delivery
UC: umbilical cord
PBMC: peripheral blood mononuclear cells
LPS: lipopolysaccharides
TNF-α: tumor necrosis factor alpha
IL-6: interleukin 6
TTTS: twin-to-twin transfusion syndrome
FETO: fetal endoscopic tracheal occlusion
