# Supplementary figures and images for: High maternal BMI and low maternal blood BDNF may determine the limit of detection of amniotic fluid BDNF throughout gestation: Analysis of mother-fetus trios and literature review
Source: PLoS One. 2022 Mar 10;17(3):e0265186. doi: 10.1371/journal.pone.0265186 (PMC8912268; doi:10.1371/journal.pone.0265186)

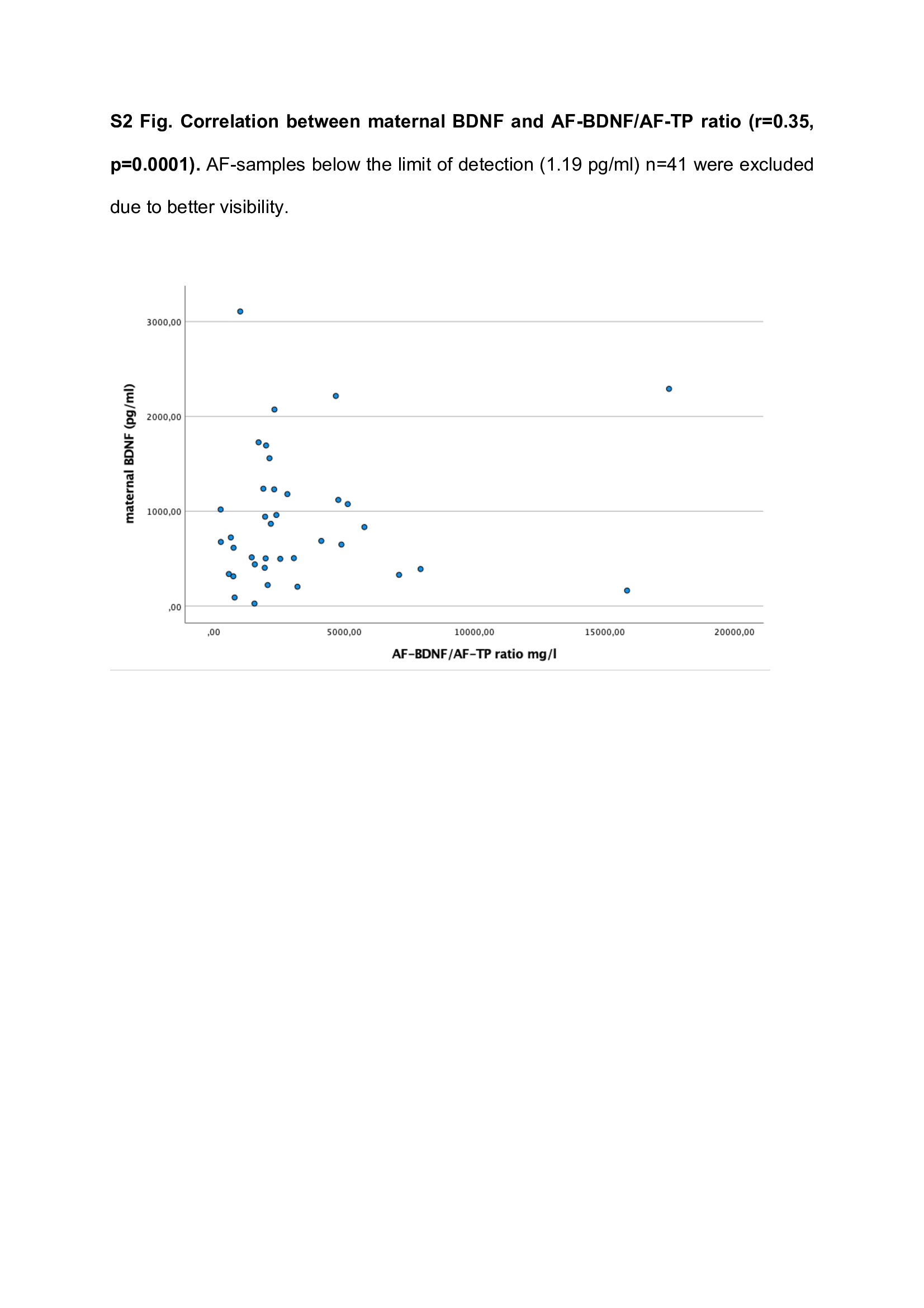

Supplement: S1 Fig — (TIFF) [file pone.0265186.s002.tiff]

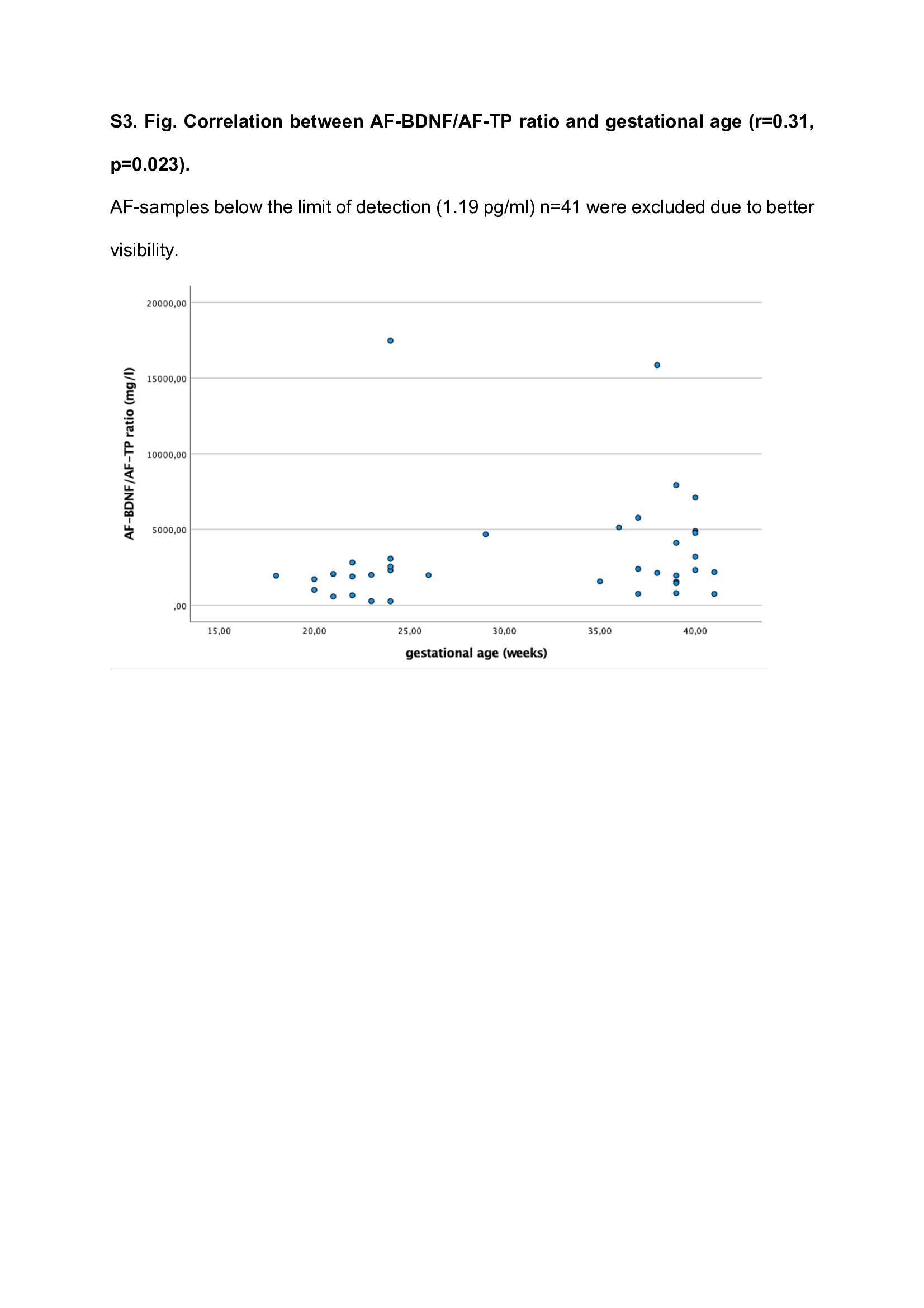

Supplement: S2 Fig — (TIFF) [file pone.0265186.s003.tiff]

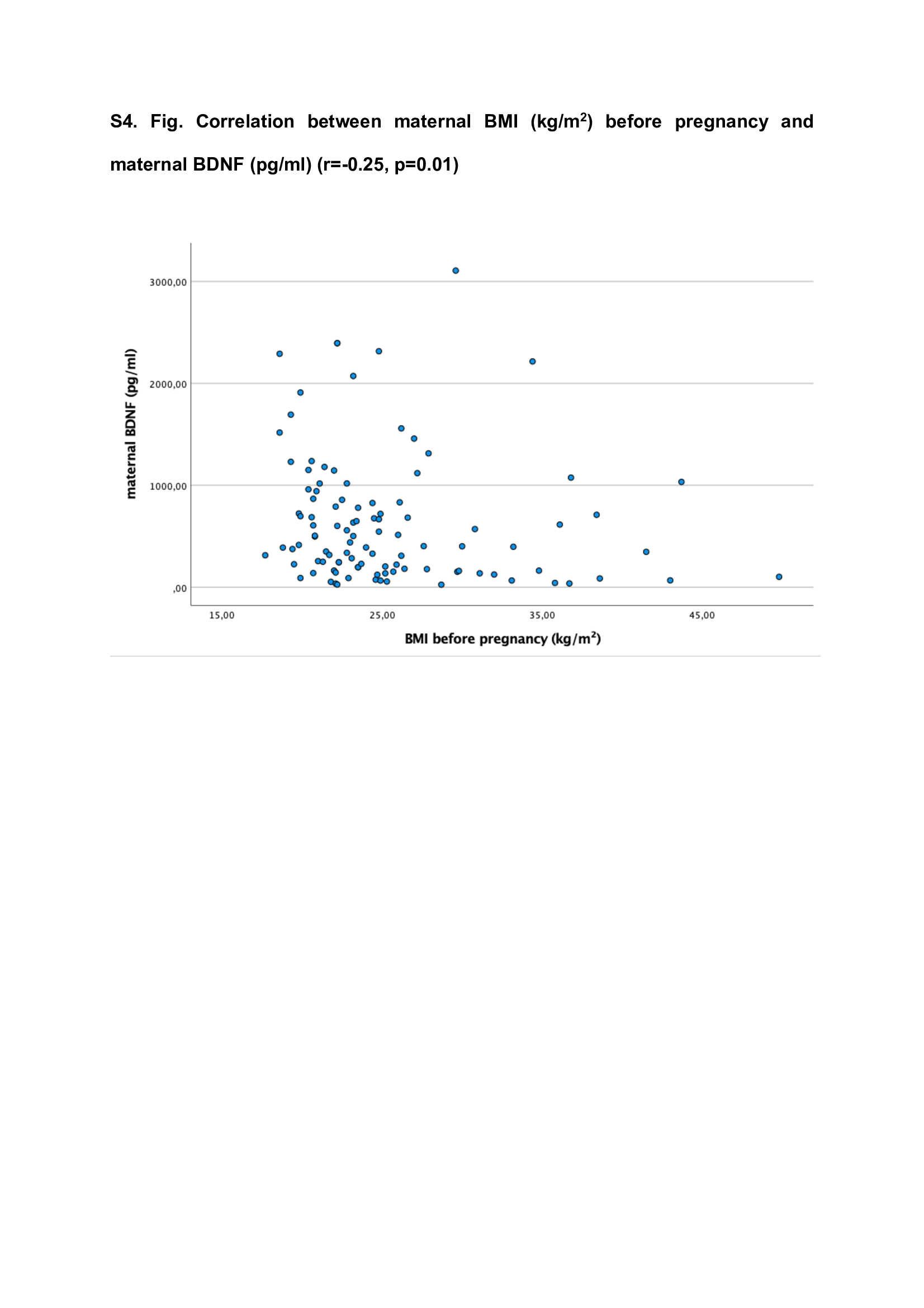

Supplement: S3 Fig — (TIFF) [file pone.0265186.s004.tiff]

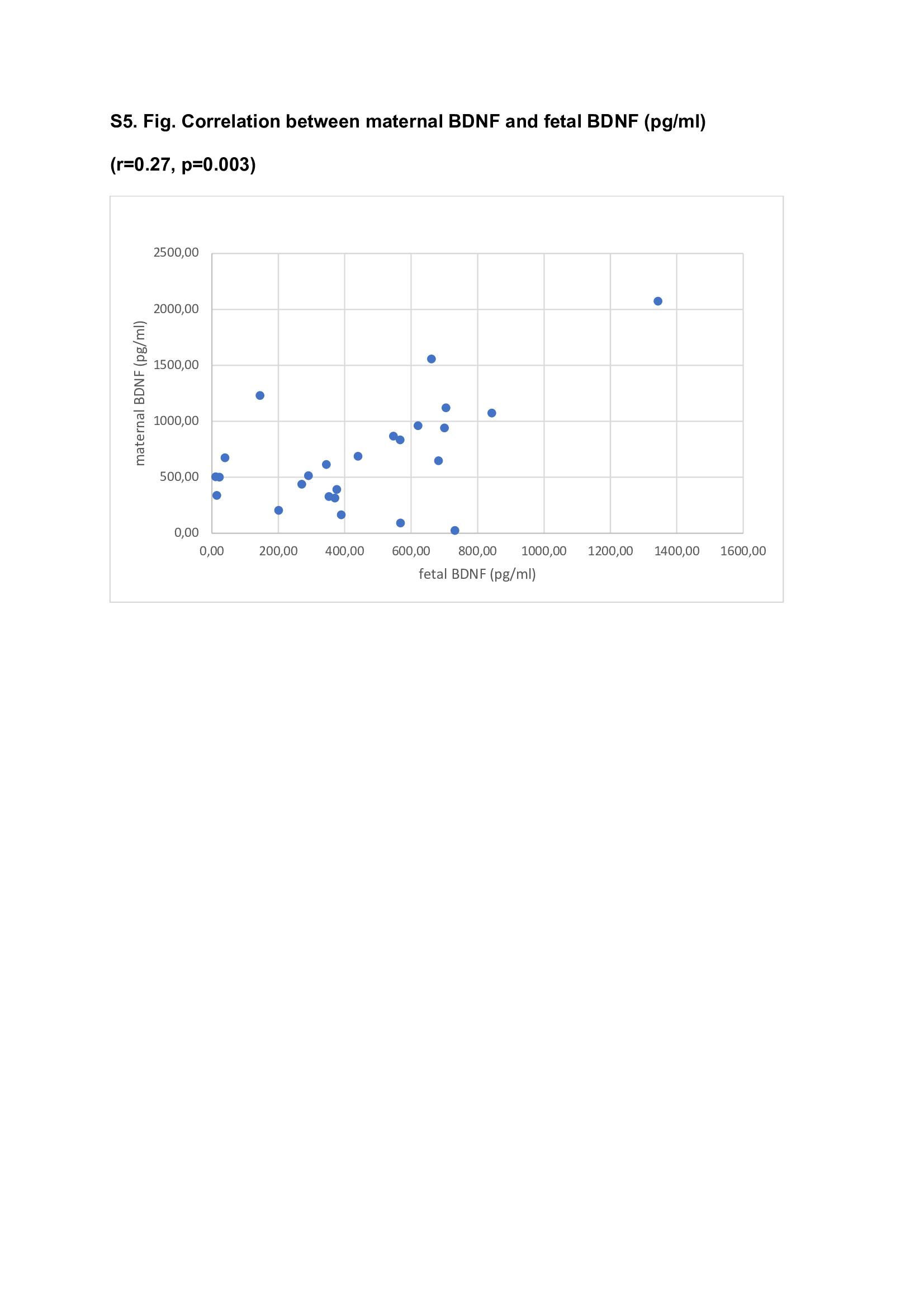

Supplement: S4 Fig — (TIFF) [file pone.0265186.s005.tiff]
